# Supplementary material for: Combined COX-2/PPARγ Expression as Independent Negative Prognosticator for Vulvar Cancer Patients
Source: Diagnostics (Basel). 2021 Mar 10;11(3):491. doi: 10.3390/diagnostics11030491 (PMC8001561; doi:10.3390/diagnostics11030491)
Supplement: Supplementary file 1 [file diagnostics-11-00491-s001.pdf]

COX-2 in non- malignant tissue of vulva **S1**

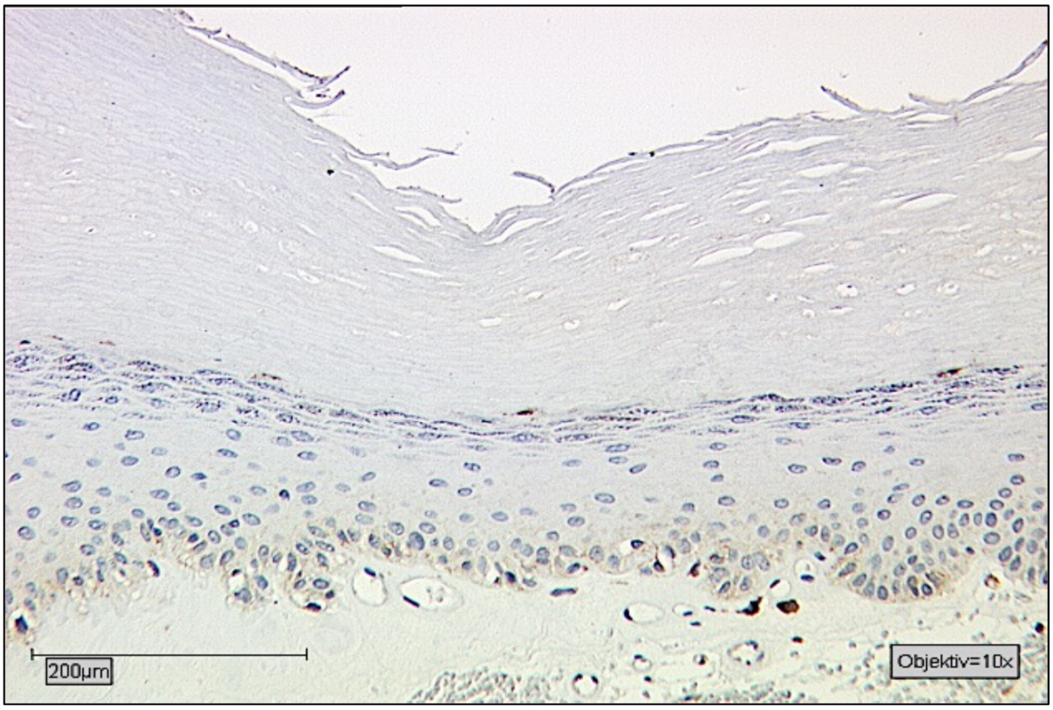

Figure S1
Immunohistochemistry staining of COX-2 (10× magnification) showing staining behavior in non-malignant vulvar tissue of patient without vulvar cancer manifestation.

PPARγ in non- malignant tissue of vulva **S2**

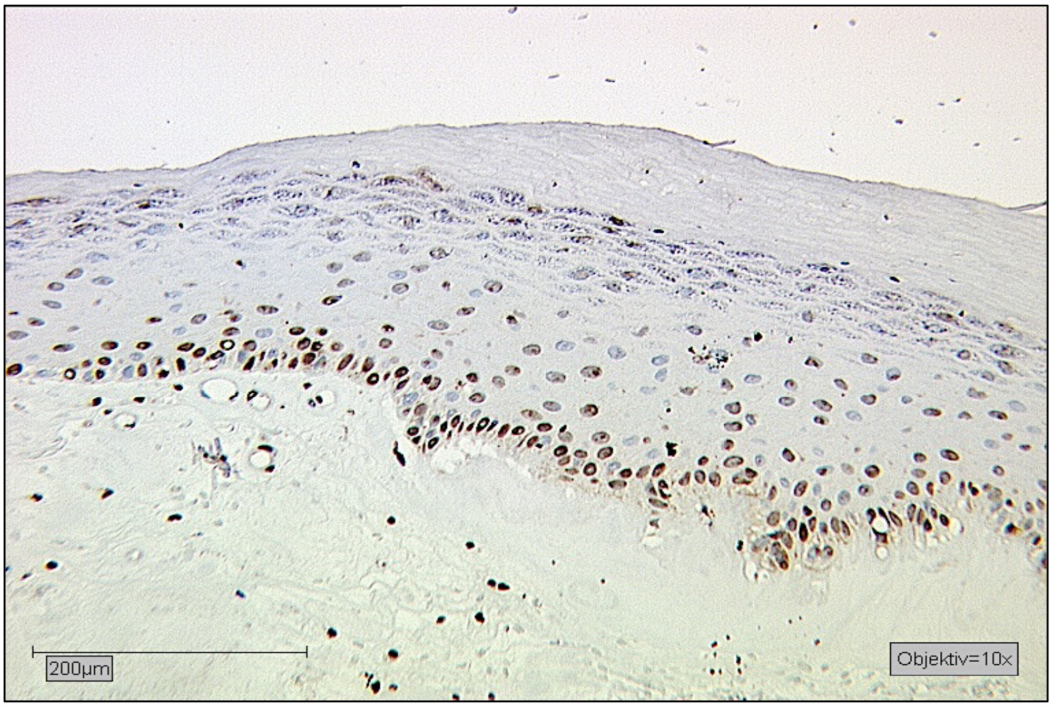

Figure S2
Immunohistochemistry staining of PPARγ (10× magnification) showing staining behavior in non-malignant vulvar tissue of patient without vulvar cancer manifestation.
